# Histones Are Passed Back to Stay in Place, More or Less

**DOI:** 10.1371/journal.pbio.1001072

**Published:** 2011-06-07

**Authors:** Richard Robinson

**Affiliations:** Freelance Science Writer, Sherborn, Massachusetts, United States of America

**Figure pbio-1001072-g001:**
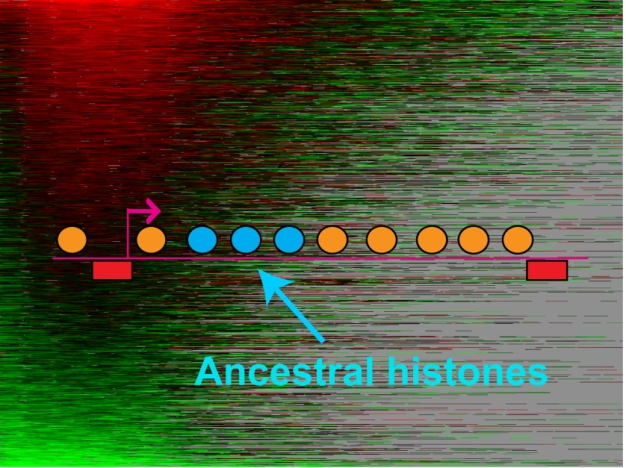
Retention of ancestral histone proteins in yeast. Tracking old histone proteins over several generations reveals a surprising accumulation of old proteins near the 5′ ends of long, poorly transcribed genes, and shows that maternal histone reincorporation during replication is imprecise.

[Fig pbio-1001072-g001]A chromosome's DNA is a single long thread, and like thread, it must be carefully spooled to keep it from becoming a hopeless tangle. In eukaryotes, that job falls to histones, proteins that link together to form barrel-shaped nucleosomes, around which DNA wraps to form the most fundamental level of chromosome structure. But histones are more than just an unvarying genomic scaffold. It is now clear that, due to the various chemical groups added to them throughout life, histones carry important “epigenetic” information that helps control expression of the genes in their neighborhood.

Such epigenetic information can even be inherited along with the genes themselves during cell division. But histones must dissociate from the DNA during both replication and transcription, temporarily separating genes from those modified histones that influence their expression. Such major disruptions to gene/histone association pose a challenge to faithful epigenetic inheritance, and it is unclear what limits they impose on the fidelity of that inheritance.

One way to gain insight into these questions is to track histones during multiple rounds of replication. In this issue of *PLoS Biology*, Marta Radman-Livaja, Oliver Rando, and Fred van Leeuwen use a novel histone-tagging technique to follow their inheritance through multiple generations, and find that ancestral histones concentrate at the upstream end of genes, a phenomenon likely enforced by their surprising one-directional movement during gene transcription.

The authors focused on movements of histone H3, a central part of the nucleosome. They began by tagging not the protein itself, but the histone gene. They incorporated two different tags into the gene, called HA and T7, with a stop codon between them. At the start of the experiment, newly formed H3 proteins carried only the HA tag, since the stop codon prevented transcription of the T7 tag. After continuous growth with only HA-tagged histones present, they treated the cells with a chemical that induced the cell to cut out the HA tag and stop signal, leaving only the T7 tag on the gene. Thus, histones produced in subsequent generations carried the T7 tag, allowing the authors to map where both the older, HA-tagged histones and the newer, H7-tagged histones were distributed in each new generation of cells.

They found that older histones tended to cluster near the 5′ end of genes (the “upstream” end that is closest to the promoter). To account for this pattern, the authors proposed a model in which histone dynamics are governed by three factors. While a cell is growing, histones turn over—that is, leave and rejoin nucleosomes. During gene transcription, surprisingly, histones are displaced from 3′ to 5′, a process the authors termed “passback.” During replication, histones spread in both directions, due to the mechanics of the replication machinery. In their model, and supported by mutation experiments that disrupted these various effects, the net accumulation of older histones at the 5′ end is largely due to passback.

This dynamic model suggests that histone-based epigenetic information cannot be inherited in a pinpoint fashion, since individual histones or whole nucleosomes are not precisely relocalized to their original locations, either after replication or after transcription. Instead, as other experiments have suggested, the minimal “unit” of epigenetic inheritance is likely to span multiple nucleosomes, enough to cancel out the noise from these three conflicting factors. Future experiments will address whether the two replicated DNA strands handle histone movements differently, and whether mutations can be identified that influence histone movements during replication.

**Radman-Livaja M, Verzijlbergen KF, Weiner A, van Welsem T, Friedman N, et al. (2011) Patterns and Mechanisms of Ancestral Histone Protein Inheritance in Budding Yeast. doi:10.1371/journal.pbio.1001075**

